# Assessment of haptic memory using somatosensory change‐related cortical responses

**DOI:** 10.1002/hbm.25165

**Published:** 2020-08-26

**Authors:** Shunsuke Sugiyama, Tomoaki Kinukawa, Nobuyuki Takeuchi, Makoto Nishihara, Toshiki Shioiri, Koji Inui

**Affiliations:** ^1^ Department of Psychiatry and Psychotherapy Gifu University Graduate School of Medicine Gifu Japan; ^2^ Department of Anesthesiology Nagoya University Graduate School of Medicine Nagoya Japan; ^3^ Department of Psychiatry Aichi Medical University Nagakute Japan; ^4^ Multidisciplinary Pain Center, Aichi Medical University Nagakute Japan; ^5^ Department of Functioning and Disability Institute for Developmental Research, Aichi Developmental Disability Center Kasugai Japan

**Keywords:** Brodmann's area 3b, magnetoencephalography, primary somatosensory cortex, sensory memory, somatosensory‐evoked magnetic field

## Abstract

Haptic memory briefly retains somatosensory information for later use; however, how and which cortical areas are affected by haptic memory remain unclear. We used change‐related cortical responses to investigate the relationship between the somatosensory cortex and haptic memory objectively. Electrical pulses, at 50 Hz with a duration of 500 ms, were randomly applied to the second, third, and fourth fingers of the right and left hands at an even probability every 800 ms. Each stimulus was labeled as D (preceded by a different side) or S (preceded by the same side). The D stimuli were further classified into 1D, 2D, and 3D, according to the number of different preceding stimuli. The S stimuli were similarly divided into 1S and 2S. The somatosensory‐evoked magnetic fields obtained were divided into four components via a dipole analysis, and each component's amplitudes were measured using the source strength waveform. The results showed that the preceding event did not affect the amplitude of the earliest 20–30 ms response in the primary somatosensory cortex. However, in the subsequent three components, the cortical activity amplitude was largest in 3D, followed by 2D, 1D, and S. These results indicate that such modulatory effects occurred somewhere in the somatosensory processing pathway higher than Brodmann's area 3b. To the best of our knowledge, this is the first study to demonstrate the existence of haptic memory for somatosensory laterality and its impact on the somatosensory cortex using change‐related cortical responses without contamination from peripheral effects.

## Graphical abstract

Electrical pulses were randomly applied to the right and left hands. The somatosensory‐evoked magnetic fields obtained were divided into four components (early SI, middle SI, late SI, and cSII). For each component, there are five event histories (1D, 2D, 3D, 1S, and 2S), which indicate the number of different (D) or similar (S) tactile stimuli that precede the probe stimulus.

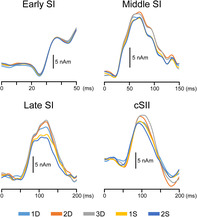

## 1 INTRODUCTION

According to the Atkinson–Shiffrin model, human memory has three separate components: a sensory register, a short‐term store, and a long‐term store (Atkinson & Shiffrin, 1968). The sensory register, or sensory memory, holds sensory information briefly for use in the next step of the multi‐store memory system. The sensory memory mechanisms underlying the visual and auditory systems have been examined for several decades (Baddeley & Hitch, 1974; Coltheart, 1980; Dick, 1974; McGaugh, 2000). Psychological studies have revealed the existence of brief storage (Brown, 1958; Waugh & Norman, 1965) and sensory memory properties, including its decay (Darwin, Turvey, & Crowder, 1972; Ricker, Vergauwe, & Cowan, 2016; Sperling, 1960), vulnerability to interference stimuli (Deutsch, 1970; Waugh & Norman, 1968), and the effects of stimulus repetition (Massaro, 1970). However, psychological studies are challenging to perform because sensory memory is outside of cognitive control and only lasts for a few seconds (Brown, 1958; Darwin et al., 1972; Glucksberg & Cowen, 1970).

In previous decades, the properties of sensory memory, particularly echoic memory, have been examined, often with mismatch negativity (MMN) paradigms (Kujala, Tervaniemi, & Schröger, 2007; Näätänen & Winkler, 1999; Picton, Alain, Otten, Ritter, & Achim, 2000; Schröger, 2007). MMN is a component of event‐related potentials (ERPs) and is elicited by a deviant stimulus, embedded in sequences of repetitive stimuli, under an oddball paradigm, with maximum negativity at Fz and positivity at the mastoid. Moreover, MMN reflects the automatic change detection process based on a short‐term memory trace. Therefore, MMN can index sensory memory (Näätänen, Jacobsen, & Winkler, 2005). For example, studies using MMN have revealed the lifetime of sensory memory (Böttcher‐Gandor & Ullsperger, 1992; Glass, Sachse, & von Suchodoletz, 2008; Mäntysalo & Näätänen, 1987; Sams, Hämäläinen, Hari, & McEvoy, 1993). However, few studies have examined MMN in the somatosensory system (Akatsuka, Wasaka, Nakata, Kida, Hoshiyama, et al., 2007; Akatsuka, Wasaka, Nakata, Kida, & Kakigi, 2007; Kekoni et al., 1997; Shinozaki, Yabe, Sutoh, Hiruma, & Kaneko, 1998), and the existence of sensory memory for somatosensory laterality has not been investigated.

We used the change‐related cortical response, which is a type of event‐related response that is specifically elicited when the brain detects sensory information that is different from the preceding sensory input (Inui et al., 2010a, 2010b, 2012, 2013; Inui, Takeuchi, Sugiyama, Motomura, & Nishihara, 2018; Kinukawa et al., 2019; Nishihara et al., 2011, 2014; Sugiyama et al., 2019; Takeuchi et al., 2019; Takeuchi, Sugiyama, Inui, Kanemoto, & Nishihara, 2017, 2018), to study sensory memory. Change‐related responses depend on a number of factors: the degree of sensory change (Inui et al., 2010b; Nishihara et al., 2011; Otsuru et al., 2011); the length of the stimulus that should be stored (Inui et al., 2010a); the length of the preceding sensory input that must be compared (Akiyama, Yamashiro, Inui, & Kakigi, 2011; Otsuru et al., 2011; Yamashiro, Inui, Otsuru, Kida, & Kakigi, 2009); the length of the decay time of the storage of previous events (Inui et al., 2010a; Urakawa, Inui, Yamashiro, Tanaka, & Kakigi, 2010; Yamashiro, Inui, Otsuru, & Kakigi, 2011); and the probability of the control and change stimuli (Inui et al., 2010b; Ohoyama et al., 2012). Thus, change‐related responses are generated based on sensory memory and comparison processes. Change‐related cortical responses and MMNs are thought to be similar components; however, no studies have compared them directly. One important difference is that change‐related cortical responses can be elicited without repetition of a frequent stimulus, whereas MMN cannot (Inui et al., 2010a, 2010b).

The current study aimed to investigate haptic memory properties using change‐related cortical responses. Haptic memory is defined as a form of sensory memory specific to tactile stimuli. Sensory memory is considered automatic and outside of cognitive control. Therefore, it is different from working memory (e.g., Katus, Grubert, & Eimer, 2015). Although iconic and echoic memories have been assessed extensively, information about haptic memory is limited (Gallace & Spence, 2009). Previous studies in both animals (Zhou & Fuster, 1996) and humans (Harris, Miniussi, Harris, & Diamond, 2002) have shown that the primary somatosensory cortex (SI) initially contributes to memory in the somatosensory system. However, the relationship between the timing and location of activation in the somatosensory cortex and memory remains unclear. Change‐related cortical responses can be distinctly observed, via electroencephalography (EEG) or magnetoencephalography (MEG), without an individual's active involvement. Therefore, these tools are useful for investigating sensory memory. Furthermore, somatosensory‐evoked magnetic fields (SEFs) can be divided into several components with distinct origins and temporal properties. Thus, we hypothesized that sensory memory is involved during some stages of somatosensory processing through SI, the secondary somatosensory cortex (SII), and the posterior parietal cortex. Such processes elicit change‐related responses and are, therefore, detectable by SEFs. To validate this hypothesis, we used a stimulation paradigm with two tactile stimuli delivered to the left and right hands at an even probability. If haptic memory works in real time, a tactile stimulus, followed by a stimulus to the different side, may evoke a change‐related cortical response. Furthermore, we assessed the differences in the stages of the somatosensory processing hierarchy.

## 2 METHODS

### 2.1 Ethics

This study was approved by the ethics committee of the National Institute for Physiological Sciences, Okazaki, Japan, and was conducted in accordance with the Declaration of Helsinki. Written informed consent was obtained from all participants before the study.

### 2.2 Participants

There were 13 healthy volunteers (four women and nine men) with a mean age of 33.4 (range: 26–56) years participating in the current study. The participants had no history of mental or neurological disorders or abuse within the previous 2 years and were not taking any medications.

### 2.3 Tactile stimulation

**FIGURE 1:**
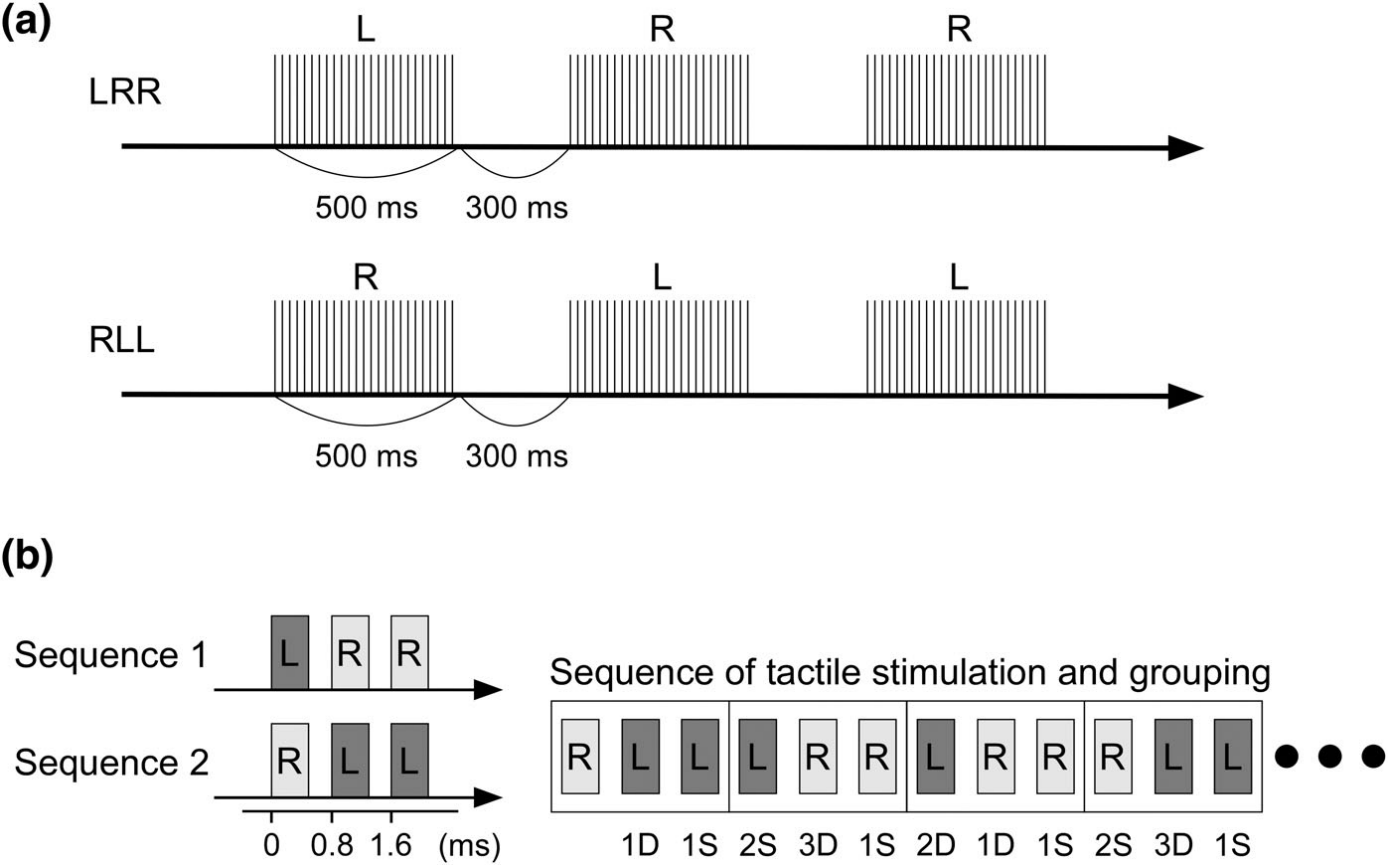
Schematic illustration of the tactile stimulation. (a) Two stimulus sequences comprising three tactile stimuli: right–left–left and left–right–right. The two sequences were repeated randomly at an even probability. For both L and R, stimuli were delivered randomly to the second, third, and fourth fingers of the respective hand. (b) Labeling of each tactile stimulus, according to preceding events (D or S) and the number of repeats (Akatsuka, Wasaka, Nakata, Kida, Hoshiyama, et al., 2007; Akatsuka, Wasaka, Nakata, Kida, & Kakigi, 2007; Akiyama et al., 2011). D, different; S, same

SEFs were elicited using a train of constant‐current square‐wave pulses of 0.5 ms at 50 Hz. The stimulus comprised 25 pulses with a total duration of 500 ms (Figure 1a). Stimuli were applied to the second, third, and fourth fingers of the right and left hands using a ring electrode, with the cathode attached to the proximal part of the finger and the anode to the distal part. Stimuli were randomly delivered to the participants' six fingers. The inter‐stimulus interval was 300 ms. The stimulus intensity was adjusted to twice the sensory threshold.

Without distinguishing between the stimulus of each finger, two stimulus sequences, composed of three tactile stimuli, were established as follows: left–right–right (LRR) and right–left–left (RLL) (Figure 1b). The three fingers on each side were stimulated randomly at an even probability; however, the same finger was not stimulated consecutively because repeated electrical pulses to the same finger have been shown to reduce the SEF responses (Grill‐Spector, Henson, & Martin, 2006; McLaughlin & Kelly, 1993; Näätänen & Picton, 1987; Thompson, 2009). The two sequences, LRR and RLL, were presented randomly at an even probability with an interval of 300 ms. Under this paradigm, the probability of each tactile stimulus (L or R) was even, and the probability of trials with changes (L to R or R to L, DIFF trials) and trials without changes (SAME trials) was even. In the DIFF trials, there were three types of events with an even probability: a trial with a stimulus preceded by a stimulus at a different side (1D) (LR and RL); a trial preceded by two stimuli at a different side (2D) (LLR and RRL); and a trial preceded by three stimuli at a different side (3D) (LLLR and RRRL). In the SAME trials, there were two types of events: a trial with a stimulus preceded by a stimulus at the same side (1S) (LL and RR) and a trial preceded by two stimuli at the same side (2S) (LLL and RRR) (Figure 1b). Therefore, this study had five types of events (1D, 2D, 3D, 1S, and 2S), with an occurrence probability of 1:1:1:2:1. None of the participants could identify the stimuli sequence.

### 2.4 Recordings

The experiments were performed in a quiet, magnetically shielded room. The participants were required to ignore tactile stimuli. To reduce their burden during the experiment, they sat in a chair and watched a silent movie on a screen placed 1.5 m in front of them. Since the visual stimulus derived from the silent movie was not time‐locked to the tactile stimuli, it is unlikely that visual‐somatosensory interaction played an important role in the present study, unlike in previous reports (e.g., Galvez‐Pol, Calvo‐Merino, & Forster, 2020).

The magnetic signals were recorded using a 306‐channel whole‐head MEG system (Vector‐view, ELEKTA Neuromag, Helsinki, Finland) comprising 102 identical triple sensor elements. Each sensor element included two orthogonal planar gradiometers and one magnetometer coupled with a multi‐superconducting quantum interference device, thereby providing three independent magnetic field measurements. The MEG signals were recorded using 204 planar‐type gradiometers that had sufficient power to detect the largest signal over local cerebral sources. Before recording, a current was fed to four head‐position indicator (HPI) coils placed at known sites to obtain the head's exact location with respect to the sensors, and the resulting magnetic fields were measured using the magnetometer. This approach allowed the individual head coordinate system to align with the magnetometer coordinate system. The four HPI coils attached to each participant's head were measured with respect to the three anatomical landmarks using a three‐dimensional digitizer. The X‐axis was fixed with the preauricular points, with right as the positive direction. The positive Y‐axis passed via the nasion, and the Z‐axis pointed upward. The signals were recorded using a bandpass filter of 0.1–300 Hz and were digitized at 4000 Hz. Epochs with MEG signals greater than 2.7 pT/cm were excluded from the averaging. The waveform was filtered digitally, with a bandpass filter of 1–100 Hz when focusing on N20 m–P30 m, and was otherwise filtered with a bandpass filter of 1–35 Hz. The analysis was conducted from 100 ms before to 400 ms after the onset of each stimulus. The 100‐ms pre‐stimulus period was used as the baseline. The average of the epochs for the four equiprobable events (1D, 2D, 3D, and 2S) were obtained at least 100 times and, therefore, around 200 times for 1S.

### 2.5 Analysis

**FIGURE 2:**
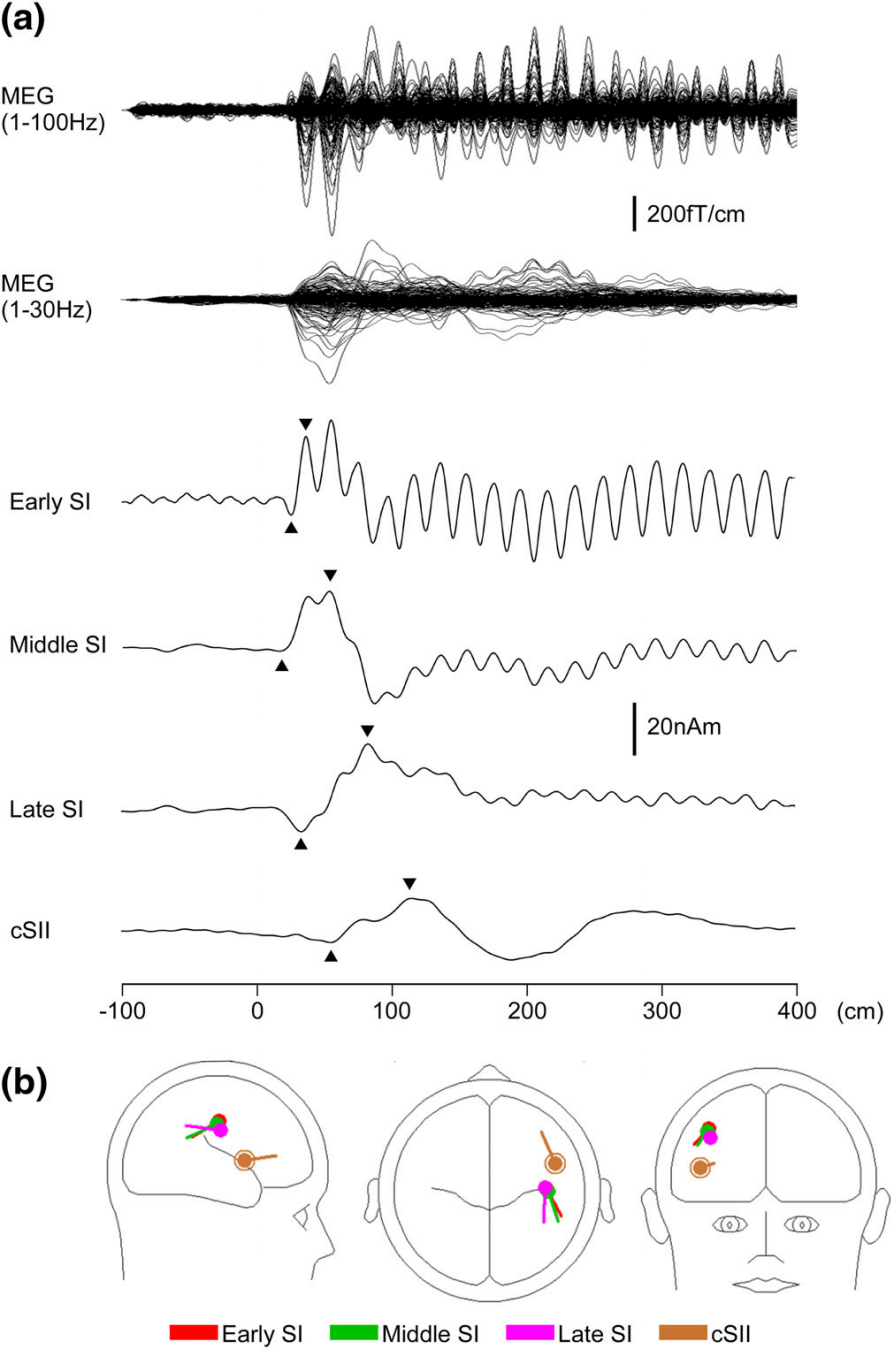
Multi‐dipole analysis. A representative participant's data after undergoing left tactile stimulation. (a) Superimposed magnetoencephalography waveforms, recorded using 204 gradiometers with a bandpass filter of 1–100 and 1–30 Hz and source strength waveforms of dipoles in early SI, middle SI, late SI, and cSII. Each waveform's measured peak‐to‐peak amplitudes are indicated by arrowheads. (b) Dipole locations and orientations of each cortical source. SI, the primary somatosensory cortex; cSII, the secondary somatosensory cortex contralateral to the stimulated side

The Brain Electrical Source Analysis software package (GmbH, Grafefling, Germany) was used to perform dipole analyses on each participant. First, all five events were added for L and R. Under a bandpass filter of 1–100 Hz and a notch filter of 59–61 Hz, the equivalent current dipole for N20/P30 m was estimated in the SI of each hemisphere, as described previously (Sugiyama, Takeuchi, Inui, Nishihara, & Shioiri, 2018). The one dipole model was then applied to waveforms for all conditions, and the source strength waveforms obtained were used to measure the amplitude of the cortical response. The peak amplitude was defined as the difference between the peak of N20 m, and the polarity reversal later peaked at around 35 ms (P30 m) (an early component generated in SI: early SI). This procedure minimizes problems caused by a baseline shift (Inui et al., 2010b; Kinukawa et al., 2019). Next, under a bandpass filter of 1–35 Hz, two dipoles located in SI and one dipole in the secondary somatosensory cortex contralateral to the stimulated side (cSII) were estimated using grand‐averaged MEG waveforms. The obtained dipole models were applied to the MEG signals for all conditions, and the peak‐to‐peak amplitude for each cortical activity was measured using the source strength waveform. The first peak was defined as the greatest response between 50 and 90 ms (a middle component generated in SI: middle SI), 80 and 150 ms (a later component generated in SI: late SI) for SI, and 85 and 145 ms for cSII, whereas the second was defined as the polarity‐reversed greatest response earlier than the first peak. One participant's original MEG and source strength waveforms are shown as an example in Figure 2. Although the dipoles in early SI and middle SI in all participants were estimated, those in late SI and cSII in a few participants were not. In addition, the dipole in the ipsilateral secondary somatosensory cortex was not estimated in several participants. Therefore, the four components (early SI, middle SI, late SI, and cSII) were used for further analysis. The components' amplitude was compared using two‐way analysis of variance (ANOVA) with stimulus side (L and R) and event history (1D, 2D, 3D, 1S, and 2S) as variables. When a significant difference was observed, the amplitude was compared between pairs using the Bonferroni adjusted *t* test. All statistical analyses were performed with the significance level set at .05.

## 3 RESULTS

**FIGURE 3:**
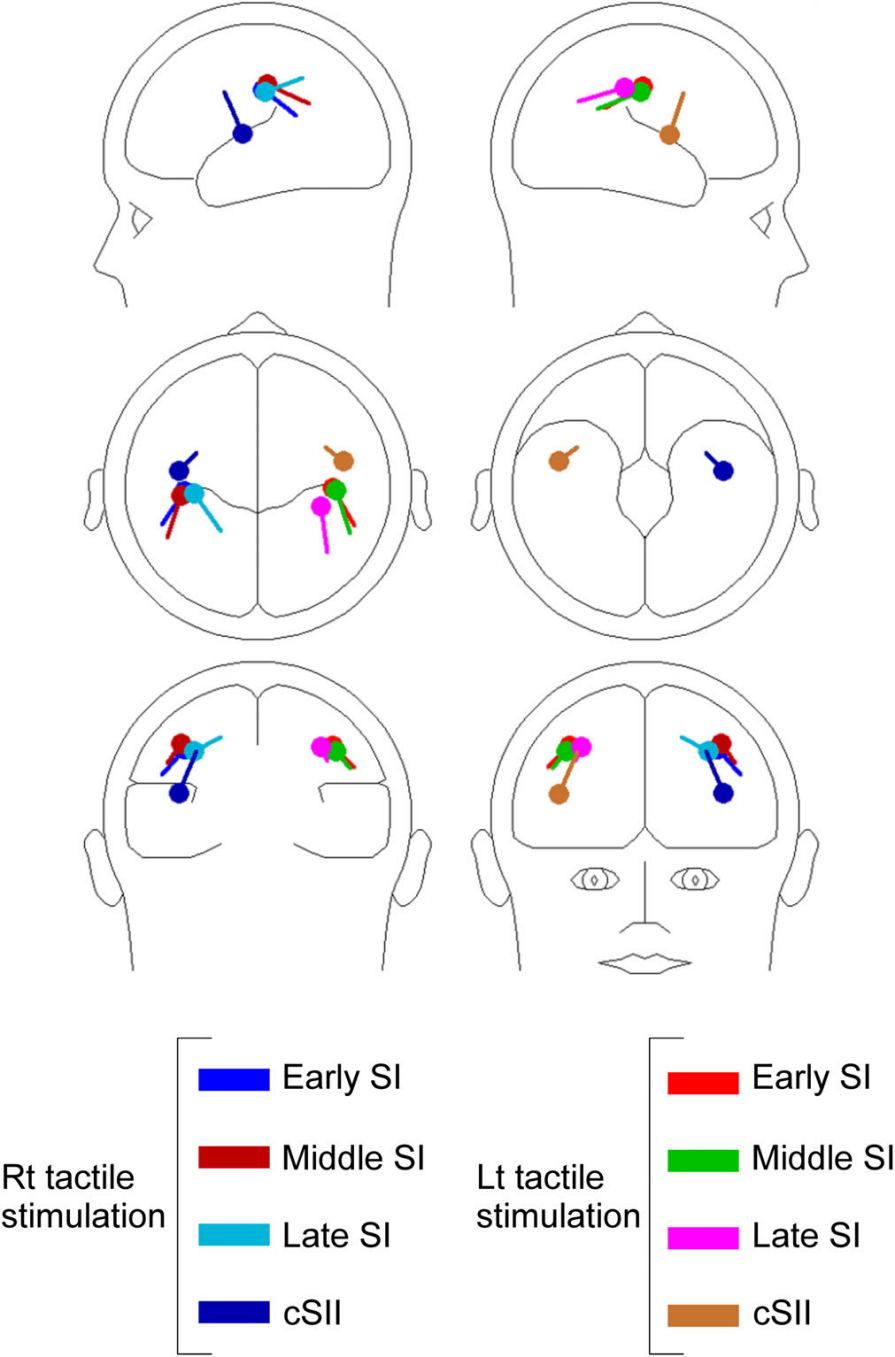
Dipole location and orientation. Mean dipole locations and orientations of each cortical source during left and right tactile stimulations. Averages across 13 subjects. The mean dipole location (circles) and orientation (bars) of each cortical source for the right (left panel) and left (right panel) side stimulation are shown. Differences between middle SI and late SI are significant for both the left (location: *p* = .014, orientation: 0.017) and right (location: *p* = .042, orientation: 0.008) stimulations. SI, the primary somatosensory cortex

**FIGURE 4:**
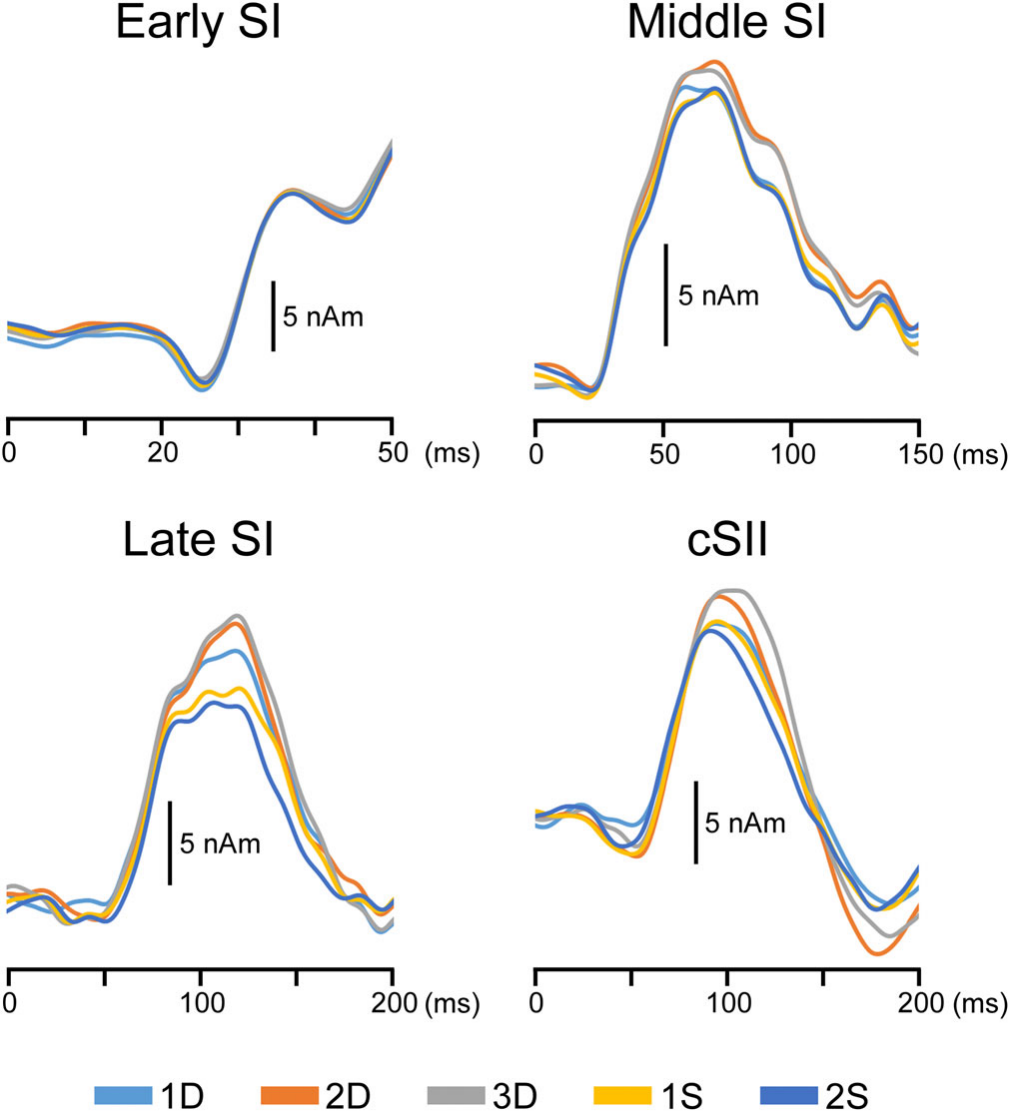
Grand‐averaged waveforms of each cortical activity. Averages across 13 subjects. For each component (early SI, middle SI, late SI, and cSII), there are five event histories (1D, 2D, 3D, 1S, and 2S), which indicate the number of different (D) or similar (S) tactile stimuli that precede the probe stimulus. Note the lack of differences among events for the early SI component (*p* = .67) but clear differences for the other three, later components (*p* < .0006). SI, the primary somatosensory cortex; cSII, the secondary somatosensory cortex contralateral to the stimulated side

Tactile stimulation elicited clear magnetic responses in the parietal and temporal areas contralateral to the stimulation, corresponding to SI and SII, respectively. Figure 3 shows the mean locations and orientations of dipoles in early SI, middle SI, late SI, and cSII. The discriminant analysis revealed that the dipoles' location and orientation did not significantly differ between early SI and middle SI for all conditions (*p* = .18–.65), whereas the differences between middle SI and late SI were significant in terms of location for both the left (*p* = .014) and right (*p* = .042) stimulations. That is, late SI was located more medially than middle SI. As shown in Figure 3, the dipole orientation was also significantly different between middle SI and late SI for both the left (*p* = .017) and right stimulations (*p* = .008). The grand‐averaged waveforms and peak amplitudes of the four components are shown in Figure 4 and Table 1, respectively. In early SI, a two‐way ANOVA revealed that both the stimulus side (*F*_1, 12_ = 0.81, *p* = .39) and event history (*F*_4, 48_ = 0.59, *p* = .67) were not significant factors in determining the amplitude. By contrast, the event histories significantly affected the amplitude of middle SI (*F*_4, 48_ = 6.46, *p* = .0003), late SI (*F*_4, 20_ = 7.76, *p* = .0006), and cSII (*F*_4, 28_ = 7.08, *p* = .0005). Meanwhile, the stimulus side (L or R) did not play a role in determining the three components' amplitude (*p* > .16). As shown in Figure 4, the 2D and 3D amplitudes were greater than those of other events in all components except early SI. For middle SI, late SI, and cSII, the mean peak amplitude was highest in 3D, followed by that in 2D, 1D, and S (Table 1). Post‐hoc tests revealed that 3D was significantly greater than 1S (middle SI: *p* = .033 and late SI: *p* = .046) and 2S (middle SI: *p* = .014, late SI: *p* = .045, and cSII: *p* = .041), except for 1S in cSII (*p* = .14). In contrast, for the other combinations of events, including 2D, no significant differences were observed based on the post‐hoc tests.

**TABLE 1:** Peak amplitude of each cortical source

	Amplitude (nAm)				
	1D	2D	3D	1S	2S
Early SI	18.5 (8.0)	17.7 (8.4)	17.7 (7.4)	18.1 (8.3)	18.3 (8.1)
Middle SI	20.0 (8.3)	20.8 (8.0)	21.6 (8.8)	19.3 (8.0)	18.7 (7.1)
Late SI	24.6 (13.7)	25.2 (15.6)	26.7 (15.5)	22.1 (13.2)	20.9 (13.7)
cSII	20.5 (11.8)	21.9 (11.8)	22.3 (11.6)	20.0 (12.0)	17.1 (11.1)

*Note:* Data are shown as mean (*SD*) values.

## 4 DISCUSSION

This study aimed to identify the existence of haptic memory for somatosensory laterality using change‐related cortical responses. Since the participants did not need to attend to, memorize, or recall the stimuli, we were able to evaluate haptic memory objectively using cortical responses. The results clearly demonstrate that presenting a single tactile stimulus shaped a memory trace that affected tactile information processing via a comparison process.

### 4.1 Methodological considerations

The stimulation paradigm was similar to that used in our recent echoic memory study (Kinukawa et al., 2019). In the present study, information was stored during presentation of a single tactile stimulus at a 500 ms duration and was used during the processing of the next tactile stimulus. This result indicates that a single event of the train of electric pulses was sufficient to retain information for later use, which is in agreement with the instantaneous nature of sensory memory. In a psychological study, a stimulus with a 25 ms duration was sufficient to establish iconic memory (Sperling, 1960). In a study using dichotic listening, a single word could be stored in memory (Glucksberg & Cowen, 1970). Although there is no similar finding about haptic memory, a single tactile stimulus with a 500 ms duration can be stored in an available form.

Under an oddball paradigm, the so‐called fresh‐afferent neuronal activity might help shape MMN (May & Tiitinen, 2010). That is, the brain must form a representation of the repetitive aspects of auditory stimulation before the occurrence of a rare stimulus, in order to elicit MMN (Näätänen et al., 2005). In this study, two tactile stimuli (L and R) were presented at an even probability. Therefore, the current study's results were unlikely based on MMN, regardless of whether the two measures have similar mechanisms. Although the possibility cannot be completely ruled out, we believe that peripheral contribution was modest in the current study with random stimulations of six digits at an even probability. The results, showing that the early SI activities in the Brodmann's area 3b did not differ in terms of amplitude in five events, clearly support this notion.

By contrast, this stimulation paradigm had a disadvantage. That is, the stimulus used each time was different even in the SAME trials. Because a change‐related cortical response is elicited whenever the brain detects differences between the current and previous sensory inputs, the responses to the S trials should, more or less, contain change‐related components, which could cause an underestimation of the difference between the D and the S trials. In fact, in a recent study examining echoic memory using a similar paradigm, the amplitude of change‐related cortical responses increased in the order of S (1S and 2S), 1D, 2D, and 3D, and a significant difference was observed between 1D and 1S (Kinukawa et al., 2019). These results demonstrate that the strength of the memory trace determined the amplitude of change‐related responses and that it was replaced with new information immediately. The current study showed a trend similar to that in our previous echoic memory study (Inui et al., 2010a, 2010b; Kinukawa et al., 2019). However, the difference between 1D and 1S was not significant, which can be attributed, at least in part, to the study's methodological weaknesses. Based on the similar effects of the preceding sensory events on the tactile and auditory change‐related responses, we believe that haptic memory and echoic memory have similar properties.

### 4.2 Memory for somatosensory processing

The initial cortical activity in SI, which is the MEG's N20 m component, reflects the excitatory post‐synaptic potentials of inputs from the thalamus to the first cortical area (Brodmann's area 3b of the somatosensory system) (Allison, Wood, McCarthy, & Spencer, 1991; Inui, Wang, Tamura, Kaneoke, & Kakigi, 2004; Wood, Cohen, Cuffin, Yarita, & Allison, 1985). N20 m increases linearly along with the stimulus intensity (Jousmäki & Forss, 1998). In the current study, we did not observe the effects of the stimulus sequence on N20 m.

In a previous study that assessed SI's physiological relevance to sensory memory using transcranial magnetic stimulation (TMS) (Harris et al., 2002), the participants were required to compare the frequencies of two vibrotactile stimuli separated by a retention interval. A pulse of TMS to SI in the retention interval was found to disrupt a participant's performance significantly. Using computational modeling, Jones, Pritchett, Stufflebeam, Hämäläinen, and Moore (2007) demonstrated that the responses evoked in SI at 70–130 ms via stimulation can predict somatosensory detection. A decrease in the latency, and an increase in the activity, of the component are correlated with tactile detection. We believe that this component corresponded to late SI observed in the current study.

In this study, the stimulus sequence affected middle SI, which has peaks at ~70 ms in the grand‐averaged waveforms. Since the cortical responses correlated to somatosensory awareness are generally considered after 100 ms (Schubert, Blankenburg, Lemm, Villringer, & Curio, 2006), the current study's results are consistent with the notion that change detection is achieved unconsciously and automatically. Electrophysiological studies have shown that selective attention enhances neural activity from SI, both in animals (Iriki, Tanaka, & Iwamura, 1996; Zhou & Fuster, 1996) and humans (Desmedt, Huy, & Bourguet, 1983; Mima, Nagamine, Nakamura, & Shibasaki, 1998). Desmedt et al. (1983) have reported that an ERP component, occurring about 30–50 ms in SI, was enhanced by attentional tasks under an oddball paradigm. Considering that attentional effects are present only after 100 ms, the study results may have reflected a certain component's enhancement under the oddball paradigm. Therefore, the SI component is likely to overlap the middle SI in the current study. Another study using EEG has investigated the adaptive properties of responses in SI and cSII over the repetitions of a stimulus (Bradley, Joyce, & Garcia‐Larrea, 2016). Adaptation is considered a mechanism that can form sensory memory (Jääskeläinen et al., 2011). Bradley et al. (2016) reported that N20 did not show significant adaptation. However, SI and cSII subsequently did. The current study's results are consistent with these findings.

### 4.3 Perspectives

In this study, we showed that haptic memory behaviors could be observed by change‐related somatosensory cortical responses. In addition, we explored, for the first time, the relationship between sensory memory and somatosensory cortical areas and activation timings in the processing hierarchy. Because haptic memory studies are limited, the present method is expected to advance haptic memory research further. Although we could not clarify each cortical area's specific role in creating sensory memory in the present study, each somatosensory cortical area has its own mechanism and role (e.g., somatotopy and laterality) in responding to salient events, suggesting that sensory memory may be involved in some sort of hierarchical sensory processing. Thus, the present method might be used to clarify the hierarchical structure of the memory system, or modification of somatosensory hierarchical processing by the memory system. Although the present study analyzed only cortical activities contralateral to the somatosensory stimulation, recent studies have reported that unilateral tactile stimulation causes bilateral representation, including in SI (e.g., Tamè, Braun, Holmes, Farnè, & Pavani, 2016). Because the subjects were required to ignore tactile stimuli in this study, ipsilateral activity was very small and difficult to identify. However, sensory memory in both hemispheres should be investigated in future research since it would reveal the significance of bilateral integration of sensory memory for spatial processing.

In clinical research using auditory MMN, sensory memory impairment has been reported, mainly in neurological and psychiatric disorders (e.g., Bartha‐Doering, Deuster, Giordano, am Zehnhoff‐Dinnesen, & Dobel, 2015). For example, patients with Alzheimer's disease (Pekkonen, Jousmäki, Könönen, Reinikainen, & Partanen, 1994), schizophrenia (Catts et al., 1995; Shelley et al., 1991), and autism spectrum disorders (Chen, Hsieh, Lin, Chan, & Cheng, 2020) have been shown to have smaller auditory MMN than normal controls. If sensory memory has a common mechanism across various sensory modalities, haptic memory may have been similarly impaired in these patients, or a certain group of patients may have a specific sensory modality deficit. However, sensory memory of other modalities is investigated less frequently in these patients, and haptic memory has not been investigated in these patients at all. The present study has also revealed somatosensory cortex areas where sensory memory contributes. Therefore, it is possible to understand the brain areas in which sensory memory is impaired, which may lead to elucidation of these diseases' pathologies. The change‐related cortical response can be elicited without repetition of a frequent stimulus, which has the advantage of a short experimental time and a low burden for the patient.

## 5 CONCLUSIONS

The presentation of a single tactile stimulus was sufficient to shape a memory trace for comparison with a subsequent, physically different tactile stimulus and to elicit change‐related scortical responses. The effects of the stimulus sequence were observed in the subsequent processes of SI and SII, but not in the earliest stage of SI. Similar to the auditory system, SI and SII work as a real‐time sensory gate open to a new event.

## CONFLICT OF INTEREST

The authors declare that the research was conducted in the absence of any commercial or financial relationships that could be construed as a potential conflict of interest.

## AUTHOR CONTRIBUTIONS

S.S., and K.I. designed the work. S.S., T.K., N.T., M.N., and K.I. performed the experiments. S.S. analyzed the data. S.S., and K.I. drafted the manuscript. T.S. commented. All authors read and approved the manuscript.

## Data Availability

Raw data were stored at the National Institute for Physiological Sciences, Okazaki, Japan. Derived data supporting the findings of this study are available from the corresponding author on request.
